# Supplementary material for: Why are some children under 24 months still undernourished in urban and peri-urban Vientiane? A mixed-methods study
Source: PLoS One. 2026 Jun 5;21(6):e0351156. doi: 10.1371/journal.pone.0351156 (PMC13240921; doi:10.1371/journal.pone.0351156)
Supplement: S1 File — (DOCX) [file pone.0351156.s001.docx]

**S1 Annex 1: Sample size calculation for quantitative study**

**1. Initial Protocol Design**: The study was originally nested within a linear programming project (the OPTIFOOD study) which aimed for a balanced recruitment of 420 mother-child pairs (210 urban and 210 peri-urban). This target was intended to provide a high level of granularity for food-based recommendation modeling.

**2. Sample Size Recalculation for Cross-Sectional Objectives**

Due to real-world implementation challenges, including underestimated census data from the Lao Statistics Bureau and caregiver unavailability during the survey period, recruitment was finalized at 333 mother-child pairs.

To ensure this reduction did not compromise the study’s ability to identify determinants of undernutrition, we performed a verification using the standard Cochran formula for cross-sectional studies:

$$n=\frac{Z^{2} p \left( 1-p \right)}{d^{2}}$$

Where:

*n* = required sample size

Z = Z-score corresponding to the desired confidence level (1.96 for 95% confidence)

*p* = estimated prevalence: 0.18 [40]

*d* = margin of error (precision), set at 5% (0.05)

$$n=\frac{{(1.96)}^{2}\times0.18\times(1-0.18)}{\left( 0.05 \right)^{2}} \approx226.8$$

The minimum required sample size to achieve statistical significance for the primary outcome was 227.

**3. Conclusion on Statistical Power**

- Our final sample of 333 pairs significantly exceeds the required minimum of 227. This provides a substantial "buffer" (an additional 106 participants), which:
- Maintains a statistical power > 80\% for identifying key risk factors.
- Allows for robust Multivariate Logistic Regression analysis (as seen in Annex 3), satisfying the requirement of having at least 10–15 outcomes per independent variable.
- Ensures that both the urban (n=166) and peri-urban (n=167) subgroups have sufficient density to allow for the comparative thematic analysis used in the qualitative phase.

**S1.1 Annex 1.1: Criteria to select the sample for the qualitative study**

The qualitative in-depth interview was collected with 3-4 fathers, 3-4 mothers, and 3-4 grandmothers in each village for 4 villages during the weekend (total of 48 caregivers), who report as best case or worst-case scenario; the randomized selection for an in-depth interview is upon the number of the population in the group, for example, the overall number of best-case is 40 persons in each village, we will do random select only 12 persons (4 father, 4 mothers, 4 grandmothers) per village.

|  | **Well nourished**  **NO Stunted and No underweight (above -2)** | Not well nourished  (stunted + underweight) |
| --- | --- | --- |
| IYCF |  |  |
| Vaccination |  |  |
| Health check-up |  |  |

**S1.2 Annex 1.2: Criteria to categorize the least well-nourished and better well-nourished**

A caregiver who has criteria of the worst case more than 50 % were categorized as worst case

| Criteria | Worst-case scenario | Best-case scenario | Note |
| --- | --- | --- | --- |
| Nutritional status | -WAZ <-2 or  - LAZ <-2 or  -WLZ <-2 | - Normal nutritional status range: - WAZ <-1 to ≥ -2 or - LAZ <-1 to ≥ -2 or - WLZ <-1 to ≥ -2 | Extract from FB questionnaire  Anthro form |
| Caregiver knowledge | *IYCF score < 5 | *IYCF score >= 5 | Extract from FB questionnaire, iycf_knowledge (A_1 to unheathyfood) |
| Vaccination | Children not received the vaccination | Children completed vaccination | Extract from FB questionnaire “form. question3.vaccard” |
| Health care check-up | Child not seen by a doctor or a health worker for a health check-up after he/she was born | child seen by a doctor or a health worker for a health check-up after he/she was born | Extract from FB questionnaire  “check-up” |
| Health problem | The child experienced any of the following health problems in the last two weeks:  - Running stomach or diarrhea/foodborne disease  - Cough or respiratory problems  - Fever | The child doesn’t have experienced any of the following health problems in the last two weeks:  - Running stomach or diarrhea/foodborne disease or  - Cough or respiratory problems or  - Fever or | Extract from FB questionnaire  “chhealthproblem” |
| Net usage | Not sleep under a bed net last night | Sleep under a bed net last night | Extract from FB questionnaire “sleepundernet” |
| Hygiene practices | Hygiene practice score <3 | Hygiene practice score >3 | Extract from FB questionnaire  “hyhiene_practice” |
| Go to ANC when the pregnant child (name) | Not go to ANC | Go to ANC | Extract from FB questionnaire  “q7_4anc” |
| times go to ANC till birth for the (name)? | Not use ANC | Use ANC at least 1 time | Extract from FB questionnaire  “q19_2” |
| Food safety and nutrition info. | Score <6 | Score >= 6 | Extract from FB questionnaire  “food_safety_and_nutrition_information”  “mchq24_1food” to “mchq24_12food” |
| Food security experience | Food insecurity in HH | Food security in HH | Extract from FB questionnaire  “Sect11” “Nofood” until “noeatwholed_freq” |

*Additional information extracted from Food based questionnaire:

**IYCF Score**:

| *10.* IYCF score | | | | |
| --- | --- | --- | --- | --- |
| **A.1** | **How often should we give the food to the non-bread fed child age 6-23 months?** | 2 time /day = 0  3 time/day =1  4 time /day =2  Don’t Know =99 | | Select <= 1 earn 1 point |
| **A.2** | **Breastfeeding is the optimal food for the child?** | Yes, strongly agree =1  Yes, agree = 2  Neutral = 3  Disagree = 4  strongly disagree = 5 | Select one of these numbers 1-3  Earn 1 point for each option | |
| **A.3** | **Exclusive breastfeeding is breastfeeding and also giving water to the child?** | Yes, strongly agree =1  Yes, agree = 2  Neutral = 3  Disagree = 4  strongly disagree = 5 | Select 4-5  Earn 1 point for each option | |
| **A.4** | **when is the suitable time to initiate the complementary feeding?** | at aged of 4 months =1  at aged of 5 months = 2  at aged of 6 months = 3  at aged of 7 months = 4  at aged of 8 months = 5  at aged of 9 months = 6  other, specify = 96 | Select one of these number 3  Earn 1 point | |
| **A.5** | **which food you can give to the child before age of 6 months?** | Not give any food nor water, only breastfeeding is enough = 0  honey/ sugar syrup =1  chew rice = 3  rice water/ rice porridge = 4  Banana = 5  fruit/ vegetable = 6  don't know = 99  other, specify =96 | Select 0  Earn 1 point | |
| **A.6** | **We should give diverse food to the child?**  **Diverse food is the various type of food such as fruit, vegetable, meat, etc.,** | Yes, strongly agree =1  Yes, agree = 2  Neutral = 3  Disagree = 4  strongly disagree = 5 | Select one of these number 1-3  Earn 1 point | |
| **A.7** | **Meat such as port, chicken, fish help the child growth properly?** | Yes, strongly agree =1  Yes, agree = 2  Neutral = 3  Disagree = 4  strongly disagree = 5 | Select one of these number 1-3  Earn 1 point | |
| **A.8** | **which food do you think is **good** for the child's health? (Can select multiple answers)** | Fruit /vegetable =1  Candy / sweet/ bakery/ice cream = 2  Soft drink/ soda = 3  Juice = 4  Breast milk substitute = 5  Breastmilk = 6  instant noodle / cane food / process food = 7  Meat/poultry/fish = 8  Rice/ cereal = 9  Egg = 10  Insect = 11  don't know = 99 | Select 1,4,5,6,8,9,10,11  Earn 1 point for each option | |
| **A.9** | **Which food do you think is **not good** for the child’s health? (Can select multiple answer)** | Fruit /vegetable =1  Candy / sweet/ bakery/ice cream = 2  Soft drink/ soda = 3  Juice = 4  Breast milk substitute = 5  Breastmilk = 6  instant noodle / cane food / process food = 7  Meat/poultry/fish = 8  Rice/ cereal = 9  Egg = 10  Insect = 11  don't know = 99 | Select 2, 3,7  Earn 1 point for each option | |

| **8. Hygiene practices** | | | | | | | |  |
| --- | --- | --- | --- | --- | --- | --- | --- | --- |
| 18.1 | Last time, before (name) ate a meal or breastfed, did the (food preparer) wash his/her hands with soap before preparing food? | | | Yes = 1  No = 2  Don’t know = 99 | | Select 1  Earn 1 point | | |
| 18.2 | Were the kitchenware and utensils used to prepare, cook and eat food washed before being used? | | | Yes = 1  No = 2  Don’t know = 99 | | Select 1  Earn 1 point | | |
| 18.3 | In the case of an infant, did the (food preparer) sterilize the feeding bottle? | | | Yes = 1  No = 2  Don’t know = 99 | | Select 1  Earn 1 point | | |
| 18.4 | Last time (name) ate, did the caregiver wash her/his hands with soap before feeding the child? | | | Yes = 1  No = 2  Don’t know = 99 | | Select 1  Earn 1 point | | |
| 18.5 | Before (name) ate the meal, did the child wash his/ her own hands with soap (or have his/her hands washed by anyone else)? | | | Yes = 1  No = 2  Don’t know = 99 | | Select 1  Earn 1 point | | |
| 18.6 | Last time you cleaned (name) after he/ she passed stool, did you wash your hands? | | | Yes = 1  No = 2 | | If no skip to 8.8 | | |
| 18.7 | How did you wash your hands after cleaning (name)? | | |  | |  | | |
|  | 1 | Water - only | |  | *You can cross (X) only one answer* | | Select 2 or 3 or 4  Earn 1 point | |
|  | 2 | Water with soap | |  |  |  |  |  |
|  | 3 | Water with ash | |  |  |  |  |  |
|  | 4 | Water with other detergent | |  |  |  |  |  |
|  | 5 | Water with soil | |  |  |  |  |  |
|  | 96 | Other (specify _______) | |  |  |  |  |  |
|  | 99 | Don’t know | |  |  |  |  |  |
| 18.8 | Last time (name) passed stool, where were the faces disposed of? | | | *You can cross (X) only one answer* | | Select 1 or 2 or 3  Earn 1 point | | |
|  | 1 | | Child used the toilet facility |  |  | |  | |
|  | 2 | | Stool was thrown in the toilet |  |  |  |  |  |
|  | 3 | | Disposed into waste/ trash |  |  |  |  |  |
|  | 4 | | Discarded outside yard |  |  |  |  |  |
|  | 5 | | Covered with soil/ ash |  |  |  |  |  |
|  | 6 | | Left where child defecated |  |  |  |  |  |
|  | 96 | | Other (specify _______) |  |  |  |  |  |
|  | 99 | | Don’t know |  |  |  |  |  |

| *Food safety and nutrition information* | | | |
| --- | --- | --- | --- |
| **24.1** | **Do you wash vegetables and fruits before eating?** | Yes = 1  No = 0 | Select 1, Earn 1 point |
| **24.2** | Do you keep the water used for cooking in a separate container/ bucket? | Yes = 1  No = 0 | Select 1, Earn 1 point |
| **24.3** | Do you eat raw fish/shellfish/meat? | Yes = 1  No = 0 | Select 0, Earn 1 point |
| **24.4** | Do you keep raw food (meat, fish, chicken, shellfish) separate from cooked food? | Yes = 1  No = 0 | Select 1, Earn 1 point |
| **24.5** | Do you use different knives to cut raw food (vegetables, meat, fish) and cooked food? | Yes = 1  No = 0 | Select 1, Earn 1 point |
| **24.6** | Do you use different cutting boards/ plates for raw food (vegetables, meat, fish) and cooked food? | Yes = 1  No = 0 | Select 1, Earn 1 point |
| **24.7** | Do you cook meat, chicken, fish, or shellfish until there is no pink meat and no blood? | Yes = 1  No = 0 | Select 1, Earn 1 point |
| **24.8** | When you make soup, do you let it cook until it steams and bubbles? | Yes = 1  No = 0 | Select 1, Earn 1 point |
| **24.9** | When you fry food (meat, fish, chicken), do you fry it in boiling oil? | Yes = 1  No = 0 | Select 1, Earn 1 point |
| **24.10** | Is cooked food still steaming when served? | Yes = 1  No = 0 | Select 1, Earn 1 point |
| **24.11** | Do you leave food at room temperature for more than 2 hours after it has been cooked? | Yes = 1  No = 0 | Select 0, Earn 1 point |
| **24.12** | Do you keep cooked and perishable food in the refrigerator? | Yes = 1  No = 0 | Select 1, Earn 1 point |
| **24.13** | Do you check the expiry date on packaged food before you eat it or use it for cooking? | Yes = 1  No = 0 | Select 1, Earn 1 point |

**Set of questionnaires to ask caregivers (Grandmothers, fathers, and mothers)**

| In-depth interview questions | Reason | | In-depth interview | | Reason | |
| --- | --- | --- | --- | --- | --- | --- |
| How many children that you have | Open the discussion | |  | |  | |
| which child do you think will need more care | To understand the importance of the care and order of the child | |  | |  | |
| How old is him (the child who the caregiver takes most care) | To know the age when check the MMF, MMD | |  | |  | |
| If the children are more than or equal to 6 months old  🡺 Did you already introduce food to the child or not? | Open discussion on food | | If the child is less than 6 months old and/or not yet introduced food and it is a mother who responds to the question please ask this set of questions instead | | | |
| You introduce complementary food when the child has how many months old? | Know the age introduce the complimentary food | | Did you already introduce food to the child or not? | | Open discussion on food | |
| What food did you give to the child yesterday? | To understand food, they eat and check the MMF, MMD | | How many hours after giving birth that you put the baby to the breast? Why? | | to check if the mother does according the SBCC guideline or not for child 0-5 month or not | |
| How many times that you give main meals (on average) to the child per day? | To know the food frequency | | What food or drink do you give to the baby after birth? why? | | to check if the mother does according to the SBCC guideline or not for children 0-5 months or not | |
| How many times that you give snacks (on average) to the child per day? | To know the food frequency | | Did you breastfeed the child with colostrum? Why? | | To know what mothers, think about colostrum | |
| What is your role for the child “name” | To understand the role and responsible for the child | | When you will stop breastfeeding (BFD)? Why? | | To understand the knowledge of the mother for BFD duration | |
| Why do you give that food to the child? | To understand the reason to give food to the child | | Do you already give water or other juice or drink or give formula milk to the child? Why | | To understand the reason why mothers not did not exclusive BFD? | |
| Who usually prepares food for the child? Why | The influence of food preparer | | Are you understand the meaning of exclusive BFD? Can you explain? | | To check the awareness of exclusive BFD | |
| How do you state which food you would like to eat | The practice of food required | | In your opinion, how do, make the child grow healthy? why? | | To understand if respondents have knowledge of healthy growth? And the answer that respondent's answer is correct or not | |
| What food do you think is good for the child, why? | To understand the knowledge on food | | How do you know if the child grows healthy? | | To understand if respondents have knowledge of healthy growth? And the answer that respondent's answer is correct or not? | |
| What food do you think is not good for the child, why? | To understand the knowledge on food | | How do you know if the child grows healthy? | | To understand if respondents have knowledge of healthy growth? And the answer that respondent's answer is correct or not? | |
| How do you know which food is good or not good for the child | To know the source of knowledge, believe of mother | | Do you vaccinate the child? why | | The health care service use of respondents and the reason | |
| How do you know that the child is like or doesn’t like the food you give? | The understanding that children like or don’t like might not be correct, Ex: the child often pushes the food that is the first taste, it is not mean that they don’t like, it is the reflection that mother should know this | | How do you know when the child gets sick? | | To know the level of care for the health of the child | |
| Can you indicate the top 5 food that children like to eat? why he/she like to eat? | Understand and interpret the mother related to food | | How do you do when the child gets sick, why? | | The health care service use of respondents and reason | |
| Can you indicate the top 5 food that a child doesn’t like to eat? why he/she like to eat? Why? | Understand and interpret of mother related to food | | If the child is less than 6 months and/or not yet introduced to food and it is the father or grandmother who responds to the question please ask this set of questions instead | | | |
| Can you indicate the food that the child should avoid? Please list as much as possible? why? | Understand the believe of food taboo | | Did you already introduce food to the child or not? | | Open discussion on food | |
| Can you indicate the food that the child should eat? Please list as much as possible? | Understand the food preference | | What is the first advice you give to the mother of the child's name …. after delivery? Why? | | Understand their advice | |
| If you have more money, which food you would like to buy? Why? | Understand the food preference when accessible or affordable | | Do you help the mother to initial BFD within 1 hour of birth? | | To check if they know that child should have BFD within 1 hours | |
| Who always decides which food to give to the child, and why? | To know who is influenced by the food given to the child | | Do you already give water or other juice or drink or give formula milk to the child? Why? | | To understand the reason why mothers do not do exclusive BFD? | |
| In your opinion, how do, make the child growth healthy? why? | To understand if respondents have knowledge of healthy growth? And the answer that respondent's answer is correct | | Are you understand the meaning of exclusive BFD? Can you explain? | | To check the awareness of exclusive BFD | |
| How do you know if the child growth healthy? | To understand if respondents have knowledge of health growth? And it is correct or not? | | Do you help to give advice to the mother to give BFD only to the child without other food or drink including water or not? | | To check the awareness of exclusive BFD | |
| How do you know if the child is not growth healthy? | To understand if respondents have knowledge of health growth? And it is correct or not? | | In your opinion, how do, make the child growth healthy? why? | | To understand if respondents have knowledge of healthy growth? And the answer that respondent's answer is correct or not? | |
| Do you vaccinate the child? why | The health care service use of respondents and reason | | How do you know if the child growth healthy? | | To understand if respondents have knowledge of health growth? And it is correct or not? | |
| How do you know when the child gets sick? | To know the level of care for the health of the child | | How do you know if the child is not growth healthy? | | To understand if respondents have knowledge of health growth? And it is correct or not? | |
| How do you do when the child gets sick, why? | The health care service use of respondents and reason | | Do you vaccinate the child? why | | The health care service use of respondents and reason | |
| What strategy did you use to encourage the child to eat more food? | Know the activity to let the child eat more food | | How do you know when the child gets sick? | | To know the level of care on health of the child | |
| What is the sign to know that the child is already hungry and you have to give food to the child? | | To know if the caregiver does not give food to the child when they are too hungry or not? | | How do you do when the child gets sick, why? | | The health care service use of respondents and reason |
| How many minutes/hours that you spend the time to play with the child? | | To compare the level of psychosocial stimulation | |  | |  |

**Additional mother questionnaires**:

| In-depth interview questions | Reason |
| --- | --- |
| What food did you eat yesterday? | To understand food, they eat |
| What is your role in the family? | To understand the role and responsibility of the mother |
| Why you taken that food? | To understand the reason to taken the food to the child |
| What food you think is good for yourself, why | To understand the knowledge on food |
| What food do you think is not good for yourself, why? | To understand the knowledge of food |
| How do you know which food is good or not good for yourself | To know the source of knowledge, believe in the mother |
| What is your most favorite food, why? | To know the food that the child likes to eat |
| Can you indicate the top 5 food that you like to eat? why do you like to eat? | Understand and interpret the mother related to food |
| Can you indicate top 5 food that you don’t like to eat? why? | Understand and interpretation of mother related to food |
| Can you indicate the food that you could avoid? Please list as much as possible? Why? | Understand the believe of food taboo |
| Can you indicate the food that you could eat? Please list as much as possible? Why? | Understand the food preference |
| If you have more money, which food you would like to buy? Why? | Understand the food preference when accessible or affordable |
| Who usually decides which food you should eat, and why? | To know the influence people to give food to the child |
| How to do when you get sick during pregnancy | The evaluate the knowledge of the mother on health care |
| What is the recommended food during pregnancy? | The evaluate the knowledge of the mother on healthy food |
| What is the restricted food during pregnancy? | The evaluate the belief on food |
| What is the recommended food after delivery? | The evaluate the knowledge of the mother on healthy food |
| What is the restricted food after delivery? | The evaluate the belief on food |
| What is the recommended food during lactating? | The evaluate the knowledge of the mother on healthy food |
| What is the restricted food during lactating? | The evaluate the belief on food |
| In your opinion, how to do it? to make you healthy? | To understand if respondents have knowledge of health growth? |
| In your opinion, what is healthy mean? | To understand if respondents have knowledge of healthy growth? And it is correct or not? |
| In your opinion, what is unhealthy mean? | To understand if respondents have knowledge of healthy growth? And it is correct or not? |
| Do you have access to Antenatal care (ANC) when pregnancy? why | The health care service use of respondent and reason |
| Do you receive the prescription from the doctor and or did you buy special vitamin during pregnancy? Why? | To know the level of care on health of the child |

**S2 Annex 2: Bivariate logistic regression - association between undernourished status and underlying risk factors among children under 24 months in two districts of Vientiane Capital (n = 333)**

|  | healthy child | | | | Stunted | | | | | Wasting | | | | | Underweight | | | | | Overweight | | | | | |  |
| --- | --- | --- | --- | --- | --- | --- | --- | --- | --- | --- | --- | --- | --- | --- | --- | --- | --- | --- | --- | --- | --- | --- | --- | --- | --- | --- |
| **Indicators** | cOR | (95%, CI) | | *P-value* | | cOR | (95%, CI) | | *P-*value | | cOR, | (95%, CI) | | *P-*value | | cOR, | 95%, CI | | *P-*value | | cOR, | | 95%, CI | | *P-value* | |
| **District** |  | Min | Max |  | |  | Min | Max |  | |  | Min | Max |  | |  | Min | Max |  | | |  | Min | Max |  | |
| Urban | 1 |  |  |  | | 1 |  |  |  | | 1 |  |  |  | | 1 |  |  |  | | | 1 |  |  |  | |
| Peri-urban | 0.673 | 0.431 | 1.052 | *0.082* | | 1.293 | 0.796 | 2.100 | *0.298* | | 0.784 | 0.266 | 2.310 | 0.658 | | 0.794 | 0.429 | 1.470 | *0.464* | | | 2.656 | 0.914 | 7.715 | *0.073* | |
| **Gender** |  |  |  |  | |  |  |  |  | |  |  |  |  | |  |  |  |  | | |  |  |  |  | |
| Girls | 1 |  |  |  | | 1 |  |  |  | | 1 |  |  |  | | 1 |  |  |  | | | 1 |  |  |  | |
| Boys | 0.493 | 0.313 | 0.778 | ***0.002*** | | 1.688 | 1.029 | 2.769 | ***0.038*** | | 3.422 | 0.937 | 12.499 | **0.063** | | 1.958 | 1.029 | 3.727 | ***0.041*** | | | 2.224 | 0.766 | 6.461 | ***0.142*** | |
| **Age in month** | | | | | | | | | | | | | | | | | | | | | | | | | |  |
| <6 | 1 |  |  |  | | 1 |  |  |  | | 1 |  |  |  | | 1 |  |  |  | | | 1 |  |  |  | |
| 6-11 | 0.909 | 0.518 | 1.596 | *0.739* | | 0.853 | 0.470 | 1.549 | *0.602* | | 1.850 | 0.430 | 7.958 | 0.409 | | 0.456 | 0.209 | 0.995 | ***0.048*** | | | 2.244 | 0.545 | 9.237 | *0.263* | |
| 11-18 | 1.565 | 0.871 | 2.812 | *0.134* | | 0.455 | 0.236 | 0.875 | ***0.018*** | | 1.850 | 0.430 | 7.958 | 0.409 | | 0.364 | 0.159 | 0.834 | ***0.017*** | | | 2.244 | 0.545 | 9.237 | *0.263* | |
| 18-24 | 1.411 | 0.639 | 3.116 | *0.394* | | 0.544 | 0.225 | 1.314 | *0.176* | | 0.935 | 0.094 | 9.281 | 0.954 | | 0.550 | 0.193 | 1.573 | *0.265* | | | 1.924 | 0.309 | 11.993 | *0.483* | |
| **Number of siblings** | | | | | | | | | | | | | | | | | | | | | | | | | |  |
| 0 | 1 |  |  |  | | 1 |  |  |  | | 1 |  |  |  | | 1 |  |  |  | | | 1 |  |  |  | |
| 1 | 0.924 | 0.508 | 1.679 | *0.795* | | 1.169 | 0.621 | 2.198 | *0.629* | | 0.798 | 0.157 | 4.057 | 0.786 | | 1.239 | 0.527 | 2.916 | 0.623 | | | 0.390 | 0.070 | 2.185 | *0.284* | |
| 2 | 0.916 | 0.487 | 1.722 | *0.785* | | 0.913 | 0.459 | 1.815 | *0.795* | | 1.025 | 0.201 | 5.226 | 0.977 | | 1.392 | 0.574 | 3.374 | 0.465 | | | 1.297 | 0.336 | 5.008 | *0.705* | |
| >=3 | 0.532 | 0.255 | 1.110 | *0.093* | | 0.783 | 0.335 | 1.830 | *0.572* | | 2.699 | 0.577 | 12.628 | 0.207 | | 1.643 | 0.599 | 4.509 | 0.335 | | | 3.154 | 0.841 | 11.822 | *0.088* | |
| ***Number of the child <5 yrs in HH*** | | | | | | | | | | | | | | | | | | | | | | | | | |  |
| 0 | 1 |  |  |  | | 1 |  |  |  | | 1 |  |  |  | | 1 |  |  |  | | | 1 |  |  |  | |
| 1 | 0.516 | 0.139 | 1.923 | *0.324* | | 2.063 | 0.446 | 9.542 | *0.354* | | 0.392 | 0.045 | 3.391 | *0.395* | | 1.927 | 0.243 | 15.283 | *0.535* | | | 0.596 | 0.071 | 4.973 | *0.632* | |
| 2 | 0.450 | 0.111 | 1.822 | *0.263* | | 2.063 | 0.408 | 10.415 | *0.381* | | 0.941 | 0.096 | 9.199 | *0.958* | | 2.348 | 0.270 | 20.388 | *0.439* | | | 0.635 | 0.060 | 6.686 | *0.705* | |
| >=3 | 0.300 | 0.054 | 1.669 | *0.169* | | 2.750 | 0.401 | 18.876 | *0.303* | | 1.091 | 0.061 | 19.630 | *0.953* | | 4.000 | 0.355 | 45.100 | *0.262* | | | (emp) |  |  |  | |
| ***Number of ther child 5-15 yrs in HH*** | | | | | | | | | | | | | | | | | | | | | | | | | |  |
| 0 | 1 |  |  |  | | 1 |  |  |  | | 1 |  |  |  | | 1 |  |  |  | | | 1 |  |  |  | |
| 1 | 0.818 | 0.495 | 1.352 | *0.433* | | 1.289 | 0.751 | 2.213 | *0.356* | | 0.988 | 0.259 | 3.763 | *0.986* | | 1.363 | 0.669 | 2.778 | *0.394* | | | 0.814 | 0.281 | 2.356 | *0.704* | |
| 2 | 0.825 | 0.429 | 1.587 | *0.564* | | 0.708 | 0.323 | 1.550 | *0.388* | | 2.367 | 0.611 | 9.174 | *0.213* | | 1.821 | 0.774 | 4.281 | *0.170* | | | 0.613 | 0.128 | 2.936 | *0.541* | |
| >=3 | 0.451 | 0.155 | 1.315 | *0.145* | | 2.601 | 0.883 | 7.665 | *0.083* | | 2.029 | 0.221 | 18.605 | *0.532* | | 1.912 | 0.490 | 7.466 | *0.351* | | | (emp) |  |  |  | |
| ***Number of adult male aged > 15 yrs in HH*** | | | | | | | | | | | | | | | | | | | | | | | | | |  |
| <=1 | 1 |  |  |  | | 1 |  |  |  | | 1 |  |  |  | | 1 |  |  |  | | | 1 |  |  |  | |
| 2 | 1.312 | 0.770 | 2.235 | *0.318* | | 0.680 | 0.377 | 1.226 | *0.200* | | 0.344 | 0.074 | 1.607 | *0.175* | | 0.412 | 0.181 | 0.938 | ***0.035*** | | | 1.967 | 0.626 | 6.185 | *0.247* | |
| >=3 adult males | 1.296 | 0.678 | 2.479 | *0.433* | | 0.748 | 0.368 | 1.518 | *0.421* | | 0.298 | 0.037 | 2.385 | *0.254* | | 0.552 | 0.217 | 1.406 | *0.213* | | | 1.165 | 0.219 | 6.213 | *0.858* | |
| >=4 adult males | 1.037 | 0.424 | 2.534 | *0.936* | | 0.812 | 0.302 | 2.181 | *0.679* | | 0.705 | 0.086 | 5.774 | *0.744* | | 0.648 | 0.181 | 2.320 | *0.505* | | | 1.985 | 0.366 | 10.771 | *0.427* | |
| ***Number of adult Female aged > 15 yrs in HH*** | | | | | | | | | | | | | | | | | | | | | | | | | |  |
| <=1 | 1 |  |  |  | | 1 |  |  |  | | 1 |  |  |  | | 1 |  |  |  | | | 1 |  |  |  | |
| 2 | 0.737 | 0.442 | 1.228 | *0.241* | | 1.265 | 0.726 | 2.205 | *0.407* | | 0.431 | 0.112 | 1.663 | *0.222* | | 0.989 | 0.474 | 2.060 | *0.975* | | | 3.048 | 0.967 | 9.607 | *0.057* | |
| >=3 | 0.692 | 0.353 | 1.358 | *0.285* | | 1.404 | 0.682 | 2.891 | *0.358* | | 0.712 | 0.146 | 3.476 | *0.674* | | 1.344 | 0.543 | 3.328 | *0.522* | | | 1.920 | 0.443 | 8.319 | *0.383* | |
| >=4 | 0.809 | 0.353 | 1.855 | *0.617* | | 0.983 | 0.386 | 2.501 | *0.971* | | 0.585 | 0.070 | 4.865 | *0.620* | | 2.139 | 0.799 | 5.723 | *0.130* | | | 1.455 | 0.162 | 13.033 | *0.738* | |
| ***'Vaccine card available in HH*** | | | | | | | | | | | | | | | | | | | | | | | | | |  |
| Yes | 1 |  |  |  | | 1 |  |  |  | | 1 |  |  |  | | 1 |  |  |  | | | 1 |  |  |  | |
| No | 0.395 | 0.146 | 1.066 | *0.067* | | 1.965 | 0.724 | 5.330 | *0.185* | | 3.378 | 0.693 | 16.467 | *0.132* | | 3.558 | 1.250 | 10.132 | *0.017* | | | (emp) |  |  |  | |
| ***Illness in the last 2 weeks*** | | | | | | | | | | | | | | | | | | | | | | | | | |  |
| **diarrhea** |  |  |  |  | |  |  |  |  | |  |  |  |  | |  |  |  |  | | |  |  |  |  | |
| No | 1 |  |  |  | | 1 |  |  |  | | 1 |  |  |  | | 1 |  |  |  | | | 1 |  |  |  | |
| Yes | (emp) |  |  |  | | (emp) |  |  |  | | (emp) |  |  |  | | 3.011 | 0.268 | 33.865 | *0.372* | | | (emp) |  |  |  | |
| **Fever** |  |  |  |  | | 1.000 |  |  |  | |  |  |  |  | |  |  |  |  | | | 1.000 |  |  |  | |
| No | 1 |  |  |  | |  |  |  |  | | 1 |  |  |  | | 1 |  |  |  | | | 1 |  |  |  | |
| Yes | 1.973 | 0.705 | 5.526 | *0.196* | | 0.835 | 0.297 | 2.349 | *0.732* | | 1.150 | 0.143 | 9.239 | *0.895* | | 0.609 | 0.137 | 2.702 | *0.514* | | | (emp) | 0.434 | 3.306 | *0.727* | |
| **cough** |  |  |  |  | |  |  |  |  | |  |  |  |  | |  |  |  |  | | |  |  |  |  | |
| No | **1** |  |  |  | | 1 |  |  |  | | 1 |  |  |  | | 1 |  |  |  | | | 1 |  |  |  | |
| Yes | 0.756 | 0.321 | 1.780 | *0.522* | | 1.483 | 0.606 | 3.627 | *0.388* | | 2.365 | 0.497 | 11.266 | *0.280* | | 0.883 | 0.252 | 3.095 | *0.846* | | | (emp) |  |  |  | |
| ***Annual HH income (unit in million kip, 1 USD = 16644.87 LAK)*** | | | | | | | | | | | | | | | | | | | | | | | | | |  |
| >50 M | 1 |  |  |  | | 1 |  |  |  | | 1 |  |  |  | | 1 |  |  |  | | | 1 |  |  |  | |
| >30 -50M | 0.818 | 0.356 | 1.881 | *0.637* | | 1.327 | 0.535 | 3.289 | *0.541* | | 1.464 | 0.128 | 16.700 | *0.759* | | 1.745 | 0.499 | 6.100 | *0.383* | | | 0.464 | 0.074 | 2.910 | *0.413* | |
| >10 -30M | 0.766 | 0.351 | 1.672 | *0.503* | | 1.193 | 0.504 | 2.827 | *0.688* | | 2.697 | 0.305 | 23.870 | *0.372* | | 1.816 | 0.553 | 5.963 | *0.325* | | | 0.500 | 0.096 | 2.593 | *0.409* | |
| > 30 -10 | 0.863 | 0.407 | 1.827 | *0.699* | | 1.106 | 0.482 | 2.537 | *0.812* | | 2.388 | 0.279 | 20.457 | *0.427* | | 1.634 | 0.513 | 5.207 | *0.406* | | | 0.757 | 0.180 | 3.177 | *0.704* | |
| 1.1–3 M | 0.900 | 0.253 | 3.197 | *0.871* | | 1.280 | 0.329 | 4.985 | *0.722* | | 1.000 |  |  |  | | 1.583 | 0.257 | 9.745 | *0.620* | | | 1.000 | 0.096 | 10.471 | *1.000* | |
| <1.1 M | 0.875 | 0.219 | 3.499 | *0.850* | | 1.829 | 0.442 | 7.557 | *0.404* | | 1.000 |  |  |  | | 2.111 | 0.333 | 13.377 | *0.428* | | | 1.000 |  |  |  | |
| ***Annual interviewee Income (unit in million kip, 1 USD = 16644.87 LAK)*** | | | | | | | | | | | | | | | | | | | | | | | | | |  |
| >50M | 1 |  |  |  | | 1 |  |  |  | | 1 |  |  |  | | 1 |  |  |  | | | 1 |  |  |  | |
| >30-50M | 1.786 | 0.436 | 7.317 | *0.420* | | 0.435 | 0.093 | 2.031 | *0.290* | | (emp) |  |  |  | | 0.185 | 0.015 | 2.273 | *0.187* | | | 1.800 | 0.401 | 8.077 | *0.443* | |
| >10-30M | 1.083 | 0.315 | 3.720 | *0.899* | | 0.836 | 0.229 | 3.054 | *0.787* | | (emp) |  |  |  | | 0.493 | 0.090 | 2.713 | *0.416* | | | 0.811 | 0.209 | 3.138 | *0.761* | |
| >30-10 | 1.362 | 0.400 | 4.634 | *0.621* | | 0.657 | 0.181 | 2.386 | *0.523* | | 1.387 | 0.300 | 6.411 | *0.675* | | 0.813 | 0.160 | 4.136 | *0.803* | | | 0.674 | 0.175 | 2.601 | *0.567* | |
| 1.1–3M | 1.735 | 0.408 | 7.368 | *0.455* | | 0.667 | 0.147 | 3.032 | *0.600* | | 1.458 | 0.337 | 6.303 | *0.613* | | 2.059 | 0.356 | 11.906 | *0.420* | | | 1.000 |  |  |  | |
| <1.1M | 1.017 | 0.297 | 3.484 | *0.978* | | 0.759 | 0.207 | 2.774 | *0.676* | | 2.333 | 0.367 | 14.852 | *0.370* | | 1.154 | 0.229 | 5.823 | *0.862* | | | 1.000 |  |  |  | |
| ***Who manage the income*** | | | | | | | | | | | | | | | | | | | | | | | | | |  |
| Parents | 1 |  |  |  | | 1 |  |  |  | | 1 |  |  |  | | 1 |  |  |  | | | 1 |  |  |  | |
| Others | 0.760 | 0.473 | 1.220 | *0.256* | | 1.107 | 0.660 | 1.854 | *0.700* | | 0.855 | 0.262 | 2.793 | *0.796* | | 2.255 | 1.211 | 4.200 | *0.010* | | | 1.543 | 0.570 | 4.173 | *0.393* | |
| ***Primary caregiver*** | | | | | | | | | | | | | | | | | | | | | | | | | |  |
| Female  HH head | 1 |  |  |  | | 1 |  |  |  | | 1 |  |  |  | | 1 |  |  |  | | | 1 |  |  |  | |
| Female non-  HH head | 0.444 | 0.195 | 1.014 | ***0.054*** | | 6.894 | 1.614 | 29.444 | *0.009* | | 1.743 | 0.599 | 0.220 | *0.599* | | 1.029 | 0.375 | 2.819 | *0.956* | | | 0.548 | 0.147 | 2.043 | *0.370* | |
| Male HH  Head | 0.245 | 0.064 | 0.939 | ***0.040*** | | 14.571 | 2.420 | 87.729 | *0.003* | | 2.917 | 0.461 | 0.169 | *0.461* | | 1.127 | 0.190 | 6.673 | *0.895* | | | 0.917 | 0.087 | 9.686 | *0.942* | |
| Grandparents | 0.508 | 0.164 | 1.578 | *0.241* | | 8.000 | 1.528 | 41.875 | *0.014* | | 1.000 | (emp) |  |  | | 1.550 | 0.397 | 6.046 | *0.528* | | | 0.458 | 0.045 | 4.680 | *0.510* | |
| Others | 1.429 | 0.145 | 14.055 | *0.760* | | 3.400 | 0.258 | 44.758 | *0.352* | | 1.000 | (emp) |  |  | | 1.000 | (emp) |  |  | | | 1.000 | (emp) |  |  | |
| ***The Food Insecurity Experience Scale*** | | | | | | | | | | | | | | | | | | | | | | | | | |  |
| No | 1 |  |  |  | | 1 |  |  |  | | 1 |  |  |  | | 1 |  |  |  | | | 1 |  |  |  | |
| Yes | 2.718 | 0.578 | 12.794 | *0.206* | | 0.380 | 0.297 | 0.486 | *<0.001* | | (emp) |  |  |  | | 0.587 | 0.073 | 4.695 | *0.616* | | | 1.875 | 0.226 | 15.562 | *0.560* | |
| ***Market in the village*** | | | | | | | | | | | | | | | | | | | | | | | | | |  |
| Yes | 1 |  |  |  | | 1 |  |  |  | | 1 |  |  |  | | 1 |  |  |  | | | 1 |  |  |  | |
| No | 1.022 | 0.648 | 1.612 | *0.924* | | 0.871 | 0.529 | 1.436 | *0.589* | | 2.153 | 0.729 | 6.355 | *0.165* | | 0.835 | 0.441 | 1.579 | *0.578* | | | 1.813 | 0.681 | 4.826 | *0.234* | |
| ***The expenditure in the last 30 days*** | | | | | | | | | | | | | | | | | | | | | | | | | |  |
| ***Food expenditure*** | | | | | | | | | | | | | | | | | | | | | | | | | |  |
| <= mean | 1 |  |  |  | | 1 |  |  |  | | 1 |  |  |  | | 1 |  |  |  | | | 1 |  |  |  | |
| > mean | 0.952 | 0.549 | 1.651 | *0.861* | | 0.945 | 0.523 | 1.709 | *0.852* | | 1.123 | 0.305 | 4.142 | *0.862* | | 0.868 | 0.413 | 1.826 | *0.710* | | | 2.039 | 0.449 | 9.257 | *0.356* | |
| ***Mean household expenditure*** | | | | | | | | | | | | | | | | | | | | | | | | | |  |
| **<= mean** | 1 |  |  |  | |  |  |  |  | | 1 |  |  |  | | 1 |  |  |  | | | 1 |  |  |  | |
| > mean | 1.224 | 0.686 | 2.183 | *0.494* | | 0.914 | 0.488 | 1.714 | *0.780* | | 0.672 | 0.147 | 3.081 | *0.609* | | 0.869 | 0.383 | 1.969 | *0.736* | | | 0.531 | 0.118 | 2.384 | *0.409* | |
| ***Child nutritional status*** | | | | | | | | | | | | | | | | | | | | | | | | | |  |
| ***Child average weight, Mean (SD)*** | | | | | | | | | | | | | | | | | | | | | | | | | |  |
| >= mean | 1 |  |  |  | | 1 |  |  |  | | (emp) |  |  |  | | 1 |  |  |  | | | 1 |  |  |  | |
| < mean | 0.374 | 0.235 | 0.596 | *0.000* | | 3.092 | 1.828 | 5.231 | ***<0.001*** | | (emp) |  |  |  | | 6.508 | 2.825 | 14.993 | ***<0.001*** | | | 0.258 | 0.082 | 0.807 | ***0.020*** | |
| ***Child average heigh, Mean (SD)*** | | | | | | | | | | | | | | | | | | | | | | | | | |  |
| >= mean | 1 |  |  |  | | 1 |  |  |  | | 1 |  |  |  | | 1 |  |  |  | | | 1 |  |  |  | |
| < mean | 0.459 | 0.292 | 0.723 | *0.292* | | 4.069 | 2.388 | 6.933 | ***<0.001*** | | 1.032 | 0.354 | 3.009 | *0.954* | | 3.680 | 1.839 | 7.364 | ***<0.001*** | | | 1.310 | 0.493 | 3.482 | *0.588* | |
| ***Mother nutritional status*** | | | | | | | | | | | | | | | | | | | | | | | | | |  |
| ***Mean weight of mother (Kg), (SD)*** | | | | | | | | | | | | | | | | | | | | | | | | | |  |
| >=mean | 1 |  |  |  | | 1 |  |  |  | | 1 |  |  |  | | 1 |  |  |  | | | 1 |  |  |  | |
| < mean | 0.592 | 0.370 | 0.947 | ***0.029*** | | 2.033 | 1.207 | 3.423 | ***0.008*** | | 1.622 | 0.478 | 5.504 | *0.438* | | 3.765 | 1.744 | 8.125 | ***<0.001*** | | | 0.459 | 0.163 | 1.296 | *0.142* | |
| ***Mean height of mother (meter)*** | | | | | | | | | | | | | | | | | | | | | | | | | |  |
| >=mean | 1 |  |  |  | | 1 |  |  |  | | 1 |  |  |  | | 1 |  |  |  | | | 1 |  |  |  | |
| < mean | 0.405 | 0.257 | 0.639 | *<0.001* | | 2.247 | 1.370 | 3.686 | *<0.001* | | 1.560 | 0.529 | 4.599 | *0.420* | | 2.634 | 1.383 | 5.017 | ***0.003*** | | | 1.310 | 0.493 | 3.482 | *0.588* | |
| ***Mother body mass index (BMI)*** | | | | | | | | | | | | | | | | | | | | | | | | | |  |
| Normal | 1 |  |  |  | | 1 |  |  |  | | 1 |  |  |  | | 1 |  |  |  | | | 1 |  |  |  | |
| Underweight | 1.360 | 0.580 | 3.187 | *0.479* | | 0.906 | 0.378 | 2.172 | ***0.825*** | | 0.827 | 0.096 | 7.160 | *0.863* | | 1.143 | 0.420 | 3.108 | ***0.794*** | | | 1.000 |  |  |  | |
| Overweight | 2.880 | 1.322 | 6.273 | ***0.008*** | | 0.272 | 0.108 | 0.687 | ***0.006*** | | 0.478 | 0.056 | 4.077 | *0.500* | | 0.381 | 0.126 | 1.155 | ***0.088*** | | | 1.182 | 0.221 | 6.310 | *0.845* | |
| Obese | 1.326 | 0.783 | 2.246 | *0.293* | | 0.565 | 0.317 | 1.007 | ***0.053*** | | 0.887 | 0.243 | 3.228 | *0.855* | | 0.344 | 0.149 | 0.794 | ***0.012*** | | | 2.543 | 0.825 | 7.837 | *0.104* | |
| ***Mother age group*** | | | | | | | | | | | | | | | | | | | | | | | | | |  |
| <=24 | 1 |  |  |  | | 1 |  |  |  | | 1 |  |  |  | | 1 |  |  |  | | | 1 |  |  |  | |
| 25-35 | 0.838 | 0.511 | 1.376 | *0.486* | | 0.931 | 0.547 | 1.584 | *0.791* | | 7.091 | 0.909 | 55.321 | *0.062* | | 0.741 | 0.383 | 1.433 | *0.374* | | | 1.006 | 0.328 | 3.083 | *0.992* | |
| >35 | 1.130 | 0.521 | 2.449 | *0.757* | | 0.726 | 0.309 | 1.706 | *0.462* | | 2.667 | 0.163 | 43.683 | *0.492* | | 0.690 | 0.238 | 2.004 | *0.496* | | | 1.622 | 0.369 | 7.125 | *0.522* | |
| ***Mother Education*** | | | | | | | | | | | | | | | | | | | | | | | | | |  |
| > College, professional or higher | 1 |  |  |  | | 1 |  |  |  | | 1 |  |  |  | | 1 |  |  |  | | | 1 |  |  |  | |
| < =High school | 0.814 | 0.473 | 1.401 | *0.458* | | 0.859 | 0.486 | 1.518 | *0.600* | | 1.802 | 0.394 | 8.238 | *0.447* | | 1.807 | 0.774 | 4.220 | *0.171* | | | 2.120 | 0.471 | 9.546 | *0.980* | |
| *Mean IYCF Knowlegde score* | | | | | | | | | | | | | | | | | | | | | | | | | |  |
| >=mean | 1 |  |  |  | | 1 |  |  |  | | 1 |  |  |  | | 1 |  |  |  | | | 1 |  |  |  | |
| < mean | 1.046 | 0.665 | 1.647 | *0.844* | | 0.919 | 0.562 | 1.505 | *0.738* | | 0.644 | 0.221 | 1.881 | *0.421* | | 0.610 | 0.330 | 1.128 | *0.115* | | | 1.613 | 0.555 | 4.689 | *0.380* | |
| *Mean food safety score* | | | | | | | | | | | | | | | | | | | | | | | | | |  |
| >=mean | 1 |  |  |  | | 1 |  |  |  | | 1 |  |  |  | | 1 |  |  |  | | | 1 |  |  |  | |
| < mean | 1.313 | 0.838 | 2.057 | *0.234* | | 0.919 | 0.562 | 1.505 | *0.738* | | 1.641 | 0.557 | 4.838 | *0.369* | | 0.927 | 0.501 | 1.717 | *0.810* | | | 0.836 | 0.310 | 2.252 | *0.723* | |
| ***Ever received health training or health education*** | | | | | | | | | | | | | | | | | | | | | | | | | |  |
| Yes | 1 |  |  |  | | 1 |  |  |  | | 1 |  |  |  | | 1 |  |  |  | | | 1 |  |  |  | |
| No | 0.345 | 0.097 | 1.227 | *0.100* | | 6.273 | 0.820 | 48.008 | ***0.077*** | | (emp) |  |  |  | | 2.796 | 0.362 | 21.584 | ***0.324*** | | | 0.853 | 0.106 | 6.844 | *0.881* | |
| ***Training on how to take care of the child after birth*** | | | | | | | | | | | | | | | | | | | | | | | | | |  |
| Yes | 1 |  |  |  | | 1 |  |  |  | | 1 |  |  |  | | 1 |  |  |  | | | 1 |  |  |  | |
| No | 4.896 | 0.605 | 39.617 | *0.137* | | (emp) |  |  |  | | (emp) |  |  |  | | (emp) |  |  |  | | | 2.406 | 0.283 | 20.426 | *0.421* | |
| *Training on Antenatal care* | | | | | | | | | | | | | | | | | | | | | | | | | |  |
| Yes | 1 |  |  |  | | 1 |  |  |  | | 1 |  |  |  | | 1 |  |  |  | | | 1 |  |  |  | |
| No | 0.235 | 0.029 | 1.930 | *0.177* | | (emp) |  |  |  | | (emp) |  |  |  | | (emp) |  |  |  | | | 0.362 | 0.042 | 3.126 | *0.356* | |
| *Training on vaccination* | | | | | | | | | | | | | | | | | | | | | | | | | |  |
| Yes | 1 |  |  |  | | 1 |  |  |  | | 1 |  |  |  | | 1 |  |  |  | | | 1 |  |  |  | |
| No | 0.364 | 0.077 | 1.714 | *0.201* | | (empty) |  |  |  | | (empty) |  |  |  | | 1.709 | 0.214 | 13.664 | *0.613* | | | 0.523 | 0.063 | 4.340 | *0.548* | |
| *Training on child care* | | | | | | | | | | | | | | | | | | | | | | | | | |  |
| Yes | 1 |  |  |  | | 1 |  |  |  | | 1 |  |  |  | | 1 |  |  |  | | | 1 |  |  |  | |
| No | 0.364 | 0.077 | 1.714 | *0.201* | | (empty) |  |  |  | | (empty) |  |  |  | | 1.709 | 0.214 | 13.664 | *0.613* | | | 0.523 | 0.063 | 4.340 | *0.548* | |

(emp) = empty

**S3.1 Annex 3.1: Multiple logistic regression analysis of healthy children**

|  | **Healthy** | | | | | | |  |
| --- | --- | --- | --- | --- | --- | --- | --- | --- |
|  | **cOR,** | | **95%, CI** | | | ***P-*value** | | |
| **Indicators** |  | **Min** | | | **Max** | |  | |
| Mother height less than the average | **0.364** | | 0.197 | 0.674 | | ***0.001*** | | |
| Boys | **0.230** | | 0.119 | 0.446 | | ***<0.001*** | | |
| Child weight less than the average | **0.323** | | 0.165 | 0.631 | | ***0.001*** | | |
| Overweigh mother | **8.337** | | 2.404 | 28.907 | | ***0.001*** | | |
| Sibling >= 3 in HH | **0.404** | | 0.168 | 0.973 | | ***0.043*** | | |
| Mother age >35 yrs | 2.221 | | 0.781 | 6.313 | | *0.134* | | |
| Mother weight less than the average | 0.536 | | 0.285 | 1.008 | | *0.053* | | |
| Annual interviewee Income, >1.100.000-3.000.000 | **4.542** | | 1.076 | 19.166 | | ***0.039*** | | |
| >=3 other ch age 5-15 yrs in HH | 0.248 | | 0.047 | 1.316 | | *0.102* | | |
| caregiver (Male household head) | 0.345 | | 0.074 | 1.594 | | *0.173* | | |
| caregiver (Grandparents) | 0.308 | | 0.066 | 1.433 | | *0.133* | | |
| Have 'fever in the last 2 weeks | 2.943 | | 0.823 | 10.525 | | *0.097* | | |
| age 18-23 months | 0.429 | | 0.141 | 1.306 | | *0.136* | | |
| food safety score less than mean | 1.594 | | 0.860 | 2.953 | | *0.139* | | |
| Household Income | 0.303 | | 0.057 | 1.610 | | *0.161* | | |

**S3.2 Annex 3.2: Multiple logistic regression analysis of stunting**

|  | **Stunted** | | | |
| --- | --- | --- | --- | --- |
|  | **cOR,** | **95%, CI** | | ***P-*value** |
| **Indicators** |  | **Min** | **Max** |  |
| Child height less than the average | **11.342** | 3.214 | 40.020 | ***<0.001*** |
| Mother height less than the average | **2.702** | 1.384 | 5.277 | ***0.004*** |
| Overweigh mother | **0.096** | 0.023 | 0.400 | ***0.001*** |
| Boys | **2.098** | 1.077 | 4.088 | ***0.029*** |
| Obese mother | 0.522 | 0.251 | 1.088 | *0.083* |
| >=3 other ch age 5-15 yrs in HH | **9.278** | 1.789 | 48.123 | ***0.008*** |
| Age 18-23 months | **6.348** | 1.285 | 31.355 | ***0.023*** |
| Caregiver (Grandparents) | **20.871** | 2.311 | 188.526 | ***0.007*** |
| Child aged 11-18 month | 3.551 | 0.976 | 12.921 | *0.054* |
| Caregiver (Male household head) | **18.650** | 1.969 | 176.632 | ***0.011*** |
| Caregiver (Mother of the ch,non-household head) | **6.245** | 1.252 | 31.162 | ***0.026*** |
| Sibling >= 3 in HH | 0.433 | 0.147 | 1.275 | *0.129* |
| One other ch age 5-15 yrs in HH | 1.720 | 0.863 | 3.428 | *0.123* |
| Annual interviewee Income, 30.000.000-50.000.000 | 0.380 | **0.109** | **1.326** | *0.129* |

**S3.3 Annex 3.3: Multiple logistic regression analysis of wasting**

|  | **Wasted** | | | |  |
| --- | --- | --- | --- | --- | --- |
|  | **cOR,** | **95%, CI** | | ***P-*value** | |
| **Indicators** |  | **Min** | **Max** |  | |
| Sibling >= 3 in HH | 3.142 | 0.799 | 12.353 | *0.101* | |
| Boys | **4.948** | 1.030 | 23.767 | ***0.046*** | |
| Mother age 25-35 | 4.546 | 0.540 | 38.260 | *0.164* | |
| No Market | 2.287 | 0.671 | 7.799 | *0.186* | |

**S3.4 Annex 3.4: Multiple logistic regression analysis of underweight**

|  | **Underweigh** | | | |  |
| --- | --- | --- | --- | --- | --- |
|  | **cOR,** | **95%, CI** | | ***P-*value** | |
| **Indicators** |  | **Min** | **Max** |  | |
| Child weight less than the average | **25.885** | 5.236 | 127.961 | ***<0.001*** | |
| Mother weigh less than the average | **10.991** | 2.998 | 40.289 | ***<0.001*** | |
| Child age 18-23 months | **14.635** | 2.121 | 100.968 | ***0.006*** | |
| Boy | **5.097** | 1.795 | 14.471 | ***0.002*** | |
| No vaccine card | **8.932** | 1.105 | 72.222 | ***0.040*** | |
| Mother height less than the average | **4.723** | 1.709 | 13.051 | ***0.003*** | |
| Annual interviewee Income, < 1.100.000 | 2.895 | 0.808 | 10.381 | *0.103* | |
| IYCF score less than the average | **0.179** | 0.061 | 0.526 | ***0.002*** | |
| Child age 6-11 months | **0.228** | 0.066 | 0.786 | ***0.019*** | |
| Annual HH Income, >3.000.000-10.000.000 | **16.605** | 1.659 | 166.149 | ***0.017*** | |
| Annual interviewee Income, >3.000.000-10.000.000 | 3.189 | 0.980 | 10.377 | *0.054* | |
| >= 3 Sibling | **5.017** | 1.281 | 19.649 | ***0.021*** | |
| 2 Sibling | **3.666** | 1.070 | 12.559 | ***0.039*** | |
| >=4 Female Adults in HH | 3.622 | 0.737 | 17.810 | *0.113* | |
| Manage HH income (Another member) | 2.378 | 0.872 | 6.483 | *0.090* | |
| Annual interviewee Income, >30.000.000-50.000.000 | 0.143 | 0.013 | 1.602 | *0.115* | |

**S3.5 Annex 3.5: Multiple logistic regression analysis of overweight**

|  | **Overweigh** | | | |
| --- | --- | --- | --- | --- |
|  | **cOR,** | **95%, CI** | | ***P-*value** |
| **Indicators** |  | **Min** | **Max** |  |
| Child height less than the average | **0.138** | 0.029 | 0.656 | ***0.013*** |
| No market | **3.523** | 1.065 | 11.653 | ***0.039*** |
| Reside in peri-urban | 3.232 | 0.968 | 10.788 | *0.057* |
| Boy | 3.653 | 0.943 | 14.151 | *0.061* |

**S4 Annex 4**: The description by caregivers of a healthy and an unhealthy child

Both groups of caregivers provided similar descriptions of healthy and unhealthy children, using five categories: disease, emotional behaviors, eating behaviors, developmental milestones, and the body composition compared to other children of the same age:

***Diseases***: The healthy child is the one who has good health status, rarely gets sick or is cured very soon when they have disease. The well-nourished caregiver also mentions that the healthy child will have pink skin, while an unhealthy child has pale skin, often gets sick, and has an allergy.

***Emotional behaviors****:* The healthy child was alert and wanted to go to play, be naughty, be fresh and sleep well, while unhealthy children were irritable, cry or seem to be sad and interested in nothing, do not play and only sleep.

***Eating behaviors*:** The healthy child will eat well, while an unhealthy child does not eat, may refuse food, eats too little, eating only food but not rice, becoming what is called “penh zang” which is a malnourished child with a big head and big belly but flat bottom and thin legs.

***Developmental milestones:*** The healthy child was the one who can eat alone, stand strongly, walk and run (not fall) compared to other children of the same age and is intelligent while an unhealthy child did not meet the milestones for their age or reach them late, cannot walk or run strongly, and cannot understand when the parents talk with them.

***Body composition****:* The healthy child is described as “*oudom somboun*,” meaning they are plump or fat, with a symmetrical body, strong muscles, big body and growing up well, while unhealthy children were thin, have weak muscles, have a big head but flat bottom, have lower weight and height and a small body size compared to other children their age; they are called “*kot*”, meaning having a small, symmetric body but short.
