# Supplementary material for: Why are some children under 24 months still undernourished in urban and peri-urban Vientiane? A mixed-methods study
Source: PLoS One. 2026 Jun 5;21(6):e0351156. doi: 10.1371/journal.pone.0351156 (PMC13240921; doi:10.1371/journal.pone.0351156)
Supplement: Attachment — Submitted filename: Response to Reviewers.docx [file pone.0351156.s004.docx]

Editor

| **Comment** | **Response** |
| --- | --- |
| **General** |  |
| 1. Please ensure that your manuscript meets PLOS ONE's style requirements, including those for file naming. The PLOS ONE style templates can be found at  <https://journals.plos.org/plosone/s/file?id=wjVg/PLOSOne_formatting_sample_main_body.pdf> and  [https://journals.plos.org/plosone/s/file?id=ba62/PLOSOne_formatting_sample_title_authors_affiliations.pdf](https://track.editorialmanager.com/CL0/https:%2F%2Fjournals.plos.org%2Fplosone%2Fs%2Ffile%3Fid=ba62%2FPLOSOne_formatting_sample_title_authors_affiliations.pdf/1/010f019b0b3f04b0-65254a8d-36b4-43f3-97c2-624939b91e3e-000000/CzU-MLaynvkFvSNWoiU1R8Rstj-Ya_0ZjeCRUIFucMM=241) | Thank you for your advice. We have updated the manuscript and file naming conventions to strictly adhere to PLOS ONE’s formatting templates. |
| 2. Please include a complete copy of PLOS’ questionnaire on inclusivity in global research in your revised manuscript. Our policy for research in this area aims to improve transparency in the reporting of research performed outside of researchers’ own country or community. The policy applies to researchers who have travelled to a different country to conduct research, research with Indigenous populations or their lands, and research on cultural artefacts. The questionnaire can also be requested at the journal’s discretion for any other submissions, even if these conditions are not met. Please find more information on the policy and a link to download a blank copy of the questionnaire here: https://journals.plos.org/plosone/s/best-practices-in-research-reporting. Please upload a completed version of your questionnaire as Supporting Information when you resubmit your manuscript. | As this study involves research in Lao PDR, we have completed the PLOS Questionnaire on Inclusivity in Global Research and included it as Supporting Information (S5 Checklist). We also added the subsection ‘Inclusivity in global research’ to the Methods section and added the following sentence: “Additional information regarding the ethical, cultural, and scientific considerations specific to inclusivity in global research is included in the Supporting Information (S5 Checklist)” in line 226 |
| 3. We note that the grant information you provided in the ‘Funding Information’ and ‘Financial Disclosure’ sections do not match.  When you resubmit, please ensure that you provide the correct grant numbers for the awards you received for your study in the ‘Funding Information’ section | We have corrected the discrepancy to ensure the grant information is consistent. The funding was supported by the EU-funded Lao Equity through Policy Analysis and Research Networks (LEARN) Program, (Number: DCI/SANTI/2014/342-306) and the Medical Committee Netherlands-Vietnam in Lao PDR (MCNV Lao PDR). |
| 4. Thank you for stating the following financial disclosure:  “The work was supported by the EU-funded LEARN Project (Number: DCI/SANTI/2014/342-306) and MCNV Lao PDR.”  Please state what role the funders took in the study.  If the funders had no role, please state: "The funders had no role in study design, data collection and analysis, decision to publish, or preparation of the manuscript."  If this statement is not correct you must amend it as needed.  Please include this amended Role of Funder statement in your cover letter; we will change the online submission form on your behalf. | The following statement will be included in the cover letter: "The funders had no role in study design, data collection and analysis, decision to publish, or preparation of the manuscript. |
| 5. Please expand the acronym “MCNV Lao PDR.” (as indicated in your financial disclosure) so that it states the name of your funders in full.  This information should be included in your cover letter; we will change the online submission form on your behalf. | We included the expanded name of the funder and of MCNV Lao PDR in the cover letter. |
| 6. Please note that funding information should not appear in any section or other areas of your manuscript. We will only publish funding information present in the Funding Statement section of the online submission form. Please remove any funding-related text from the manuscript. | All funding-related text has been removed from the main body of the manuscript. |
| 7. If the reviewer comments include a recommendation to cite specific previously published works, please review and evaluate these publications to determine whether they are relevant and should be cited. There is no requirement to cite these works unless the editor has indicated otherwise. | We have carefully reviewed the reviewer comments for any recommendations to cite specific previously published works. We note that no specific publications were recommended by the reviewers or the editor. However, we have independently updated our reference list to include recent, relevant literature on urban nutrition in Southeast Asia to further strengthen the discussion of our findings. |
| 8. Please review your reference list to ensure that it is complete and correct. If you have cited papers that have been retracted, please include the rationale for doing so in the manuscript text, or remove these references and replace them with relevant current references. Any changes to the reference list should be mentioned in the rebuttal letter that accompanies your revised manuscript. If you need to cite a retracted article, indicate the article’s retracted status in the References list and also include a citation and full reference for the retraction notice. | We have conducted a thorough review of the reference list to ensure accuracy and completeness. We cross-checked all cited works against the Retraction Watch database and the NLM PubMed 'retracted publication' filter. We confirm that no retracted papers are cited in this manuscript. Furthermore, we have verified that all references are formatted according to the PLOS ONE 'Vancouver' style, and all URLs/DOIs provided in the references are active and correct. Any minor typographical corrections to the reference list made during this revision have been highlighted in the 'Revised Manuscript with Track Changes' file. |
|  |  |

Reviewer #1

| **Comment** | **Response** |
| --- | --- |
| **Methodology** |  |
| the research combined a quantitative survey of 333 mother-child pairs with qualitative interviews of 47 caregivers to explore underlying factors. The findings revealed that food insecurity was not the primary issue; instead, the key determinants were caregiver-related behaviors, such as over-reliance on health services for nutrition information, failure to translate knowledge into practice, and the detrimental influence of other caregivers (like grandmothers) whose traditional beliefs often prioritized filling the child's stomach over providing adequate nutritional diversity. | Thank for your appreciation of our work. |

Reviewer #2

| **Comment** | **Response** |
| --- | --- |
| **Introduction and methods:** |  |
| 1. The rationale for selecting only one urban and one peri-urban district should be more explicitly justified, as it might affect the generalizability. | The study aimed to explore factors underlying persistently high rates of undernutrition among young children in urban and peri-urban districts of Vientiane Capital in Lao PDR. We randomly selected one district representing the urban area (Saysettha) and one representing the peri-urban area (Pakgeum), located more than 60 km from the city center, as they represent the rapid nutritional transition occurring in Vientiane capital. We acknowledge in the limitations section that, due to time constraints and limited budget, the study was conducted in only two districts, which were selected randomly to represent the two areas. The findings are intended to provide valuable insights applicable to similar urbanizing contexts across Low- and Middle-Income Countries (LMICs).We have added this justification to the Methods section, line 76 |
| 2. Please specify, how the data saturation was ensured. | For the qualitative component, we conducted semi-structured interviews with 47 caregivers. Data saturation was monitored during transcription and initial open coding; recruitment was finalized when no new themes related to the study aims emerged from the last three interviews. |
| 3. Reducing the sample size reduces power of the study; So, did authors ensure if the sample size remained sufficient. | We have provided a technical justification in Annex 1. Using the standard Cochrane formula with a conservative prevalence estimate from LSIS III, the required minimum sample was 227. Our final recruitment of 333 exceeds this by over 46%, ensuring the study maintains robust statistical power (>80%) for both the bivariate and multivariate analyses presented in the Results. |
| 4. Type of sampling not mentioned. | We conducted a cross-sectional study using sequential exploratory mixed methods. For the quantitative phase, districts were randomly selected, and villages were selected based on the number of children. For the qualitative phase, preliminary quantitative data were used to identify households with either well-nourished or the least well-nourished children (a form of purposive sampling based on criteria outlined in Annex 1.1 and 1.2), and then caregivers in these households were randomly selected for semi-structured interviews. |
| **Results and Discussion:** |  |
| 1. Reliability of mothers' self-recorded diet history should be discussed. | We agree that the reliability of maternal-reported dietary data is a critical consideration. In the revised manuscript, we have added a discussion regarding the reliability of the 7-day food tally and 24-hour recall methods used. To enhance reliability, we employed trained enumerators to conduct face-to-face interviews and used standardized food portion images from the food photobook (line 109) to assist mothers in recalling intake. While we acknowledge the risk of over-reporting 'healthy' foods due to social desirability bias, the 7-day period was chosen specifically to capture a more stable representation of the child's typical diet compared to a single 24-hour recall. We have now addressed this in the 'Limitations' and 'Discussion' sections, line 645. |
| 2. Potential recall bias and social desirability bias should be acknowledged. | We agree and have now explicitly acknowledged these potential biases in the Strengths and Limitations section, line 654. We have discussed how maternal recall may be subject to recall bias regarding exact food frequencies over the 7-day period and how social desirability bias may have influenced caregivers to report practices that align with health recommendations found in the national maternal and child health 'pink' handbooks. We have also detailed the mitigation strategies used, such as non-judgmental interviewing techniques and the use of food photobooks to minimize these effects in line 661. |
| 3. Whether inter-examiner reliability measured? Or is the data collected by a single examiner? | While data were collected by a team of maternal and child health nurses rather than a single examiner, we ensured high inter-examiner reliability through a rigorous standardization protocol. Prior to data collection, all examiners had three days of training (two days on the questionnaire and one on anthropometry); one more day was used to pretest the form. To ensure accuracy in food identification and portion size estimation, a standardized food photobook was used to guide participants during the interviews on how to use the food photo book, the data underwent a dedicated one-day anthropometry orientation focused on standardized SECA measurement techniques. To minimize inter-observer variation, we utilized a 'lead-assistant' model where the nurse performed the measurement and a trained data collector cross-verified the reading before it was electronically recorded. Additionally, equipment was calibrated daily, and senior researchers conducted random spot-checks during the first week of the survey to ensure consistency across the different examiners. These procedures were implemented to ensure that the nutritional status data remained robust and comparable across all study sites. The explanation text as added under the field procedures of the methodology section, line 114. |
| 4. Qualitative study: Classification bias is not discussed. Coding and software used are not specified. | We have revised the Qualitative Data Analysis section to include the specific software and coding procedures used at line 209. To address classification bias, we utilized a 'triangulation' approach where two researchers independently coded the first subset of transcripts. Any discrepancies in theme categorization were discussed until a consensus was reached, ensuring that the findings were grounded in the participants' actual responses rather than researcher bias. We utilized ATLAS.ti version 8 to manage the data and ensure a transparent audit trail of the coding process. |
| 5. Add a more explicit statement of what new insights this study adds. | We have strengthened the Conclusion sections to more clearly articulate the novel contributions of this study at line 674. While previous research in Lao PDR has focused on rural poverty among children under five years old, this study provides new insights into the 'urban paradox' of unexpected undernutrition among children under two years of age. It highlights how, in transitioning peri-urban environments, child undernutrition persists not due to a lack of food availability, but due to shifting social dynamics. There is not yet very much reported on this situation which is increasingly common in other low- and middle-income country (LMCIs). This mixed-methods evidence offers a unique perspective on how urban transition creates specific vulnerabilities that require tailored public health interventions. |
