# Supplementary material for: Why are some children under 24 months still undernourished in urban and peri-urban Vientiane? A mixed-methods study
Source: PLoS One. 2026 Jun 5;21(6):e0351156. doi: 10.1371/journal.pone.0351156 (PMC13240921; doi:10.1371/journal.pone.0351156)
Supplement: Attachment — Submitted filename: Response to Reviewers100526.docx [file pone.0351156.s005.docx]

Editor

| **Comment** | **Response** |
| --- | --- |
| **General** |  |
| Please include the following items when submitting your revised manuscript:  • A letter that responds to each point raised by the academic editor and reviewer(s). You should upload this letter as a separate file labeled 'Response to Reviewers'.  • A marked-up copy of your manuscript that highlights changes made to the original version. You should upload this as a separate file labeled 'Revised Manuscript with Track Changes'.  • An unmarked version of your revised paper without tracked changes. You should upload this as a separate file labeled 'Manuscript'. | Thank you for your advice. We have updated the manuscript and file naming conventions to strictly adhere to PLOS ONE’s advices. |

Reviewer #3

| **Comment** | **Response** |
| --- | --- |
|  |  |
| The revised manuscript shows substantial improvement in methodological clarity and analytical depth. The authors have addressed the previous concerns thoughtfully. I appreciate the clearer explanation of sampling procedures. Importatly, the explanation of how potential data reliability concerns were identified and mitigated significantly enhances the methodological transparency of the study. I believe the manuscript in its current form makes a meaningful contribution to the literature on child undernutrition in rapidly urbanizing settings. Overall the manuscript is suitable for publication. | Thank for your appreciation of our work. |

Reviewer #4

| **Comment** | **Response** |
| --- | --- |
| **Major comments:** |  |
| This manuscript looks at an important public health issue, which is child undernutrition in urban and peri-urban areas. This is a valuable topic because most studies focus more on rural settings. The mixed methods approach is a strength and helps in understanding caregiving practices beyond just food availability. | We sincerely thank you for the thoughtful and constructive comments. We have carefully revised the manuscript to improve the methodological clarity, transparency of analysis, interpretation of findings, and presentation of the mixed-methods approach. |
| Major comments 1: Study design and mixed methods | |
| The authors should explain more clearly why this type of mixed methods approach was chosen. It is also not very clear how the qualitative findings support or add to the quantitative results. There is a long gap between the two phases of data collection. The authors should discuss whether this could affect the findings, especially due to seasonal changes in diet or food access. | Thank you for this important comment. We have revised the manuscript to clarify that we used a sequential explanatory mixed-methods approach rather than a sequential exploratory mixed-methods approach in **Lines 25, 94, and 690**. We also clarified the rationale for using the sequential explanatory mixed-methods approach, as the quantitative phase was conducted first to identify patterns and factors associated with child undernutrition, while the qualitative phase was designed to further explore caregiving practices, beliefs, and contextual factors that could help explain the quantitative findings. This clarification has been added in **Line 94**.  We acknowledge the nine-month gap between the quantitative data collection phase (23 January 2019 to 17 March 2019) and the qualitative data collection phase (18 December 2019 to 23 December 2019). However, both periods occurred during the dry season in Lao PDR, which may have reduced seasonal variation in food availability. Although seasonal food availability can influence dietary patterns, particularly in rural settings, the study was conducted in urban and peri-urban areas where food access is generally more stable throughout the year due to market availability. This is also supported by our food security findings, which showed a relatively low prevalence of household food insecurity. We have added a paragraph in the Discussion section acknowledging that seasonal availability of certain foods (e.g., specific fruits and vegetables) may still have influenced dietary recall. In addition, the qualitative component primarily explored long-term caregiving practices, beliefs, and knowledge application, which are likely to be more stable over time than short-term seasonal food availability in **Line 690.** |
| Sampling and representativeness Only one urban and one peri-urban district were included. The authors should explain how well these represent the larger population. The addition of more villages due to initial underestimation is understandable, but it may introduce some selection bias and should be discussed. | Thank you for this valuable observation. We clarified that the selected districts were intended to represent urban and peri-urban nutritional transition settings within Vientiane Capital rather than the entire Lao PDR population in **Line 79**. We also added a discussion of the potential selection bias introduced by expanding village catchment areas due to the underestimation of the eligible population in **Line 668**. |
| Statistical analysis The statistical methods are generally appropriate, but important details are missing. The authors should explain how variables were selected for the regression analysis. It is also not clear whether multicollinearity and model fit were tested. Converting variables into two groups based on the mean may reduce the quality of the analysis and should be justified. | Thank you for this valuable comment. We have revised the statistical analysis section to clarify the procedures used for variable selection and model assessment. Variables included in the multiple logistic regression models were selected based on theoretical relevance, findings from previous literature, and statistical significance in the bivariate analysis. A manual backward model reduction approach was then used to identify the final models. We also clarified that model assumptions, potential confounding among associated factors, and overall model fit were carefully evaluated during model development using the Hosmer–Lemeshow goodness-of-fit test and Receiver Operating Characteristic (ROC) curve analysis. In addition, we acknowledged the limitations of dichotomizing continuous variables based on the mean, while noting that this approach was used to facilitate interpretation and comparison between groups in this exploratory analysis. These clarifications have been added in **Lines 146–156.** |
| Definitions and classification The definition used for well-nourished children does not fully match standard WHO criteria. This needs to be clarified and justified. The method used to define overweight should also be explained more clearly. | Thank you for this important comment. We have revised the manuscript to clarify the operational definition of “well-nourished” children used in this study. Children were classified as well-nourished if they did not meet the criteria for stunting, underweight, wasting, or overweight according to the anthropometric indicators applied in this study. We acknowledge that this operational classification was developed specifically for comparative analysis and may not fully reflect the broader WHO conceptual definition of child nutritional status.  We also clarified the definition of overweight in the revised manuscript. In this study, overweight was operationally defined as Weight-for-Age Z-score (WAZ) > +2 standard deviations for analytical purposes. We acknowledge that Weight-for-Length Z-score (WHZ/WLZ) is more commonly recommended by WHO for assessing overweight among children under five years, and this limitation has now been acknowledged in the revised manuscript. These clarifications have been added in the method section, **Line 169**, and the discussion section, **Line 668.** |
| Measurement and bias The study depends partly on mothers recalling and recording dietary information. This can lead to recall bias and reporting bias. Although some steps were taken to reduce this, it should be discussed more clearly. More details are also needed on how consistency between different data collectors was maintained. | Thank you for this important point. We expanded the Methods section to provide additional details on interviewer training, standardization procedures, supervision, use of food photobooks, and anthropometric measurement protocols to reduce inter-observer variability and recall bias. We also added acknowledgment of residual recall and reporting bias in the limitations section, **Line 681.** |
| Qualitative component The qualitative part adds value, but more details are needed. The authors should explain how coding was done, how many people were involved, and how consistency was ensured. It would also help to clearly show how qualitative and quantitative findings were combined. | Thank you for this constructive suggestion. Thematic analysis was conducted using ATLAS.ti version 8. Two researchers independently reviewed transcripts to develop a preliminary codebook and resolved discrepancies through consensus-building. The qualitative findings were integrated to explain why high knowledge scores (quantitative) did not always translate to better nutritional outcomes, we have already address this in the data management and analysis **Line 225**  . |
| Interpretation of findings Some conclusions seem stronger than what the data can support, especially since this is a cross-sectional study. The authors should be more careful in suggesting cause and effect. | We agree and have revised the results and conclusion to emphasize associations and correlations rather than direct causality.  . |
| Data availability The data availability statement is not very clear. The authors should clearly state whether the full dataset is publicly available or explain any restrictions. | The anonymized dataset supporting the findings of this study is available from the corresponding author upon reasonable request and with approval from the National Ethics Committee for Health Research due to participant confidentiality considerations |
| Minor comments:  The manuscript is generally understandable, but the language can be improved for better clarity. Some sentences are long and can be simplified. There are also a few minor grammatical errors that should be corrected. Some parts of the manuscript can be shortened to avoid repetition.  Overall, this is an important study with good potential, but it needs revisions to improve clarity, methods, and interpretation. | We carefully reviewed the manuscript and revised several sentences for clarity, grammar, and conciseness. Repetitive wording was reduced and language was improved throughout the manuscript. |
