# Supplementary material for: Why are some children under 24 months still undernourished in urban and peri-urban Vientiane? A mixed-methods study
Source: PLoS One. 2026 Jun 5;21(6):e0351156. doi: 10.1371/journal.pone.0351156 (PMC13240921; doi:10.1371/journal.pone.0351156)
Supplement: Attachment — Submitted filename: Reviewer comments PONE.docx [file pone.0351156.s002.docx]

This study addresses a critical gap, on why undernutrition persists in urban areas despite food availability along with a Mixed-methods approach that enriches interpretation.

Introduction and methods:

1. The rationale for selecting only *one* urban and *one* peri-urban district should be more explicitly justified, as it might affect the generalizability.
2. Please specify, how the data saturation was ensured.
3. Reducing the sample size reduces power of the study; So, did authors ensure if the sample size remained sufficient.
4. Type of sampling not mentioned.

Results and Discussion:

1. Reliability of mothers' self-recorded diet history should be discussed.
2. Potential recall bias and social desirability bias should be acknowledged.
3. Whether inter-examiner reliability measured? Or is the data collected by a single examiner?
4. Qualitative study: Classification bias is not discussed. Coding and software used are not specified.
5. Add a more explicit statement of what new insights this study adds.
